# Perfusion Defects and Collateral Flow Patterns in Acute Small Subcortical Infarction: a 4D Dynamic MRI Study

**DOI:** 10.1007/s12975-021-00953-x

**Published:** 2021-10-14

**Authors:** Yen-Chu Huang, Jiann-Der Lee, Yi-Ting Pan, Hsu-Huei Weng, Jen-Tsung Yang, Leng-Chieh Lin, Yuan-Hsiung Tsai

**Affiliations:** 1 Department of Neurology, Chang Gung Memorial Hospital at Chiayi, Chang-Gung University College of Medicine, Chiayi County, Taiwan; 2 Department of Diagnostic Radiology, Chang Gung Memorial Hospital at Chiayi, Chang-Gung University College of Medicine, Chiayi County, Taiwan; 3 Department of Neurosurgery, Chang Gung Memorial Hospital at Chiayi, Chang-Gung University College of Medicine, Chiayi County, Taiwan; 4 Department of Emergency Medicine, Chang Gung Memorial Hospital at Chiayi, Chang-Gung University College of Medicine, Chiayi County, Taiwan

**Keywords:** Perfusion defect, Small subcortical infarction, Small vessel disease, MRI, Early neurological deterioration

## Abstract

The hemodynamic changes of acute small subcortical infarction (SSI) are not well understood. We evaluate the hemodynamic changes and collaterals in acute SSI using perfusion magnetic resonance imaging (MRI). A total of 103 patients with acute SSI in penetrating artery territories were recruited and underwent MRI within 24 h of stroke onset. Using 4D dynamic perfusion MRI, they were divided into three patterns: 25 (24%) with normal perfusion, 31 (30%) with compensated perfusion, and 47 (46%) with hypoperfusion. The development of anterograde or retrograde collaterals was also evaluated. Patients with hypoperfusion pattern had the highest rate of early neurological deterioration (32%, *p* = 0.007), the largest initial and final infarction volumes (*p* < 0.001 and *p* = 0.029), the lowest relative cerebral blood flow (0.63, *p* < 0.001), and the lowest rate of anterograde and retrograde collaterals (19%, *p* < 0.001; 66%, *p* = 0.002). The anterograde collaterals were associated with higher relative cerebral blood volume (0.91 vs. 0.77; *p* = 0.024) and a higher rate of deep cerebral microbleeds (48 vs. 21%; *p* = 0.028), whereas retrograde collaterals were associated with higher systolic and diastolic blood pressure (*p* = 0.031 and 0.020), smaller initial infarction volume (0.81 vs. 1.34 ml, *p* = 0.031), and a higher rate of lobar cerebral microbleeds (30 vs. 0%; *p* = 0.013). Both anterograde and retrograde collaterals may play a critical role in maintaining cerebral perfusion and can have an impact on patient clinical outcomes. Further studies are warranted to verify these findings and to investigate effective treatments.

**Supplementary Information:**

The online version contains supplementary material available at 10.1007/s12975-021-00953-x.

## Introduction

Clinically evident recent small subcortical infarctions (SSI), commonly called lacunar infarcts, account for around one quarter of all cases of ischemic stroke and are also one of the neuroimaging features of small vessel disease (SVD) [1]. Recent SSIs are associated with the occlusion of penetrating arteries from different pathologies of SVD, including cerebral amyloid angiopathy and hypertensive arteriopathy [2]. However, there are other pathologies including embolism, microatheroma, and branch atheromatous disease (BAD) [3, 4]. BAD is caused by occlusion or stenosis at the origin of the large caliber penetrating arteries due to microatheromas or junctional plaques [5].

In the past, lacunar infarction was thought to be caused by the occlusion of a terminal penetrating artery, without collateral circulation and the occurrence of penumbra [6, 7]. However, previous studies of postmortem human brain revealed anastomoses of the major perforators and precapillary arterioles [8, 9]. Small perfusion defects were observed in computed tomography (CT) and magnetic resonance imaging (MRI) perfusion studies and they were shown to be associated with the development of early neurological deterioration (END) [10–12], and interpreted as indicating the existence of penumbra. Nevertheless, it is questionable whether the penumbra hypothesis could be applied in the microcirculation. If we assume the hypothesis is valid, collateral circulation may be observed and plays a critical role in maintaining perfusion in the penumbral region of acute SSI.

Forster et al. first observed anterograde and retrograde collaterals in acute lacunar infarction using 4D dynamic susceptibility contrast (DSC) MR perfusion [13]. Rudilosso et al. used CT perfusion to demonstrate that there was delayed compensatory collateral supply in recent SSI [14]. However, they did not show clinically significant differences between the collateral flow patterns. Nevertheless, a recent study revealed that stroke patients with lacunar infarcts may also benefit from pharmacological reperfusion [15]. These results reinforce the notion that perfusion patterns may play a critical role in clinical interventions for patients with acute stage SSI.

In the present study, we aimed to evaluate the perfusion and collateral flow patterns in patients with acute SSI, and to evaluate their clinical significance.

## Methods

### Patients

The current study was retrospective and all patients were recruited from previous prospective studies that used MRI to predict END at Chang Gung Memorial Hospital [16]. All examinations were performed after written informed consent was obtained from the patient. We recruited patients who (1) had a clinical diagnosis of ischemic stroke without thrombolytic or thrombectomy therapy, (2) underwent a complete MRI protocol within 24 h after the onset of stroke, and (3) had a visible ischemic lesion with an axial diameter ≤ 20 mm, located in the striatocapsular, thalamic, or brain stem areas as determined by diffuse weighted imaging (DWI). Patients with relevant occlusion or > 50% stenosis of the large vessels, as identified by MR angiogram, were excluded from the study.

We recorded patient data, including age, sex, and a medical history of hypertension, diabetes mellitus, hyperlipidemia, coronary artery disease, atrial fibrillation, and prior cerebrovascular disease. Systolic and diastolic blood pressure values, cell counts, blood biochemistry and coagulation profiles, as determined on the patient’s admission, were also recorded. Neurological deficits were evaluated prospectively using the National Institutes of Health Stroke Scale (NIHSS) scores. END was defined as an elevation of ≥ 2 points on the NIHSS within 72 h of stroke onset. Clinical outcomes at 3 months were evaluated using the modified Rankin scale (mRS).

### MRI Protocol

MRI was performed using a 3 Tesla Siemens Verio MRI system (Siemens Medical System, Erlangen, Germany) with a 16-channel head coil. A standard protocol was used for all patients including (1) axial T1-magnetization prepared rapid gradient echo (MPRAGE) images, (2) axial T2-weighted turbo spin-echo (TSE) images, (3) axial diffusion-weighted images (DWI), (4) axial fluid-attenuated inversion recovery (FLAIR) images, (5) T2*-weighted gradient echo images, (6) 3D time-of-flight MR angiography, and (7) dynamic susceptibility contrast perfusion weighted images.

The imaging sequences and parameters were as follows: DWI (repetition time [TR]/echo time [TE] = 8400/90, 160 × 160 acquisition matrix, 4.0 mm slice thickness, 28 slices, b-factor = 1000 and 230 mm field of view [FOV]), axial T2-weighted TSE images (TR/TE = 3300/86 ms, 256 × 194 acquisition matrix, 4.0 mm slice thickness and 28 slices), FLAIR sequence (TR/TE = 9000/85 ms, inversion time [TI] = 2500 ms, 256 × 165 acquisition matrix, 4.0 mm slice thickness, 28 slices and 230 mm FOV), T2*-weighted gradient echo (TR/TE = 3500/2.87 ms, excitations = 1, 5.2 mm slice thickness, 18 slices, 256 × 146 matrix, 1.2 mm gap and 20° flip angle), and MPRAGE images (TR/TE = 3500/2.87 ms, TI = 1100 ms, 256 × 256 acquisition matrix, 9° flip angle, 1.0 mm slice thickness). The MR angiography used 3D time of flight (TR/TE = 21/3.6 ms and 0.6 mm thickness) covering the extracranial carotid artery and the circle of Willis. The dynamic susceptibility contrast perfusion weighted imaging scans were acquired using a gradient echo echo-planar imaging sequence (TR = 1500 ms, TE = 36 ms, FOV = 220 mm, matrix = 92 × 92, 17 × 6-mm slices, and scan time = 1 min 36 s) with an intravenous bolus injection of gadolinium contrast agent (0.2 mmol/kg) at the fifth dynamic [17].

### Postprocessing and Image Analysis

MIStar (Apollo Medical Imaging Technology, Melbourne, Australia) was used to generate the perfusion imaging for each patient. The automatic arterial input function (AIF) was used and then adjusted manually if the quality of the automatic AIF was not satisfactory. Standard singular value decomposition was used to generate quantitative cerebral blood flow (CBF), cerebral blood volume (CBV) and mean transit time (MTT) maps.

The topographic location of the acute infarction was first identified on DWIs. With reference to the location of the acute infarction, the location of the perfusion delay was identified visually using the MTT maps. A region of interest (ROI) was placed covering the hypoperfused area on the MTT maps whenever a well-defined hypoperfused area was identified. Alternatively, a ROI was placed on the acute infarction in the DWI maps and it was then shifted into the perfusion maps. The relative MTT (rMTT) ratio was calculated as the ratio of the ROI value of the lesion side to the contralateral normal hemisphere. The ROIs were shifted to the CBV and CBF maps to calculate the relative CBV (rCBV) and relative CBF (rCBF) ratios, respectively.

All of the images were evaluated by two experienced stroke neurologists, both of whom were blinded to the clinical information. Any results with discrepancies were reviewed again by both neurologists until a consensus was reached.

#### DSC Perfusion Imaging

The DSC perfusion imaging was obtained from perfusion-weighted raw images in each frame to demonstrate the sequential visualization of blood flow in a 1.5-s interval, from the baseline prebolus image to the late venous phase. The perfusion defect on the DSC perfusion imaging was traced with reference to the topographical location of the perfusion defect in the MTT maps or the acute infarction in the DWI maps.

**Fig. 1:**
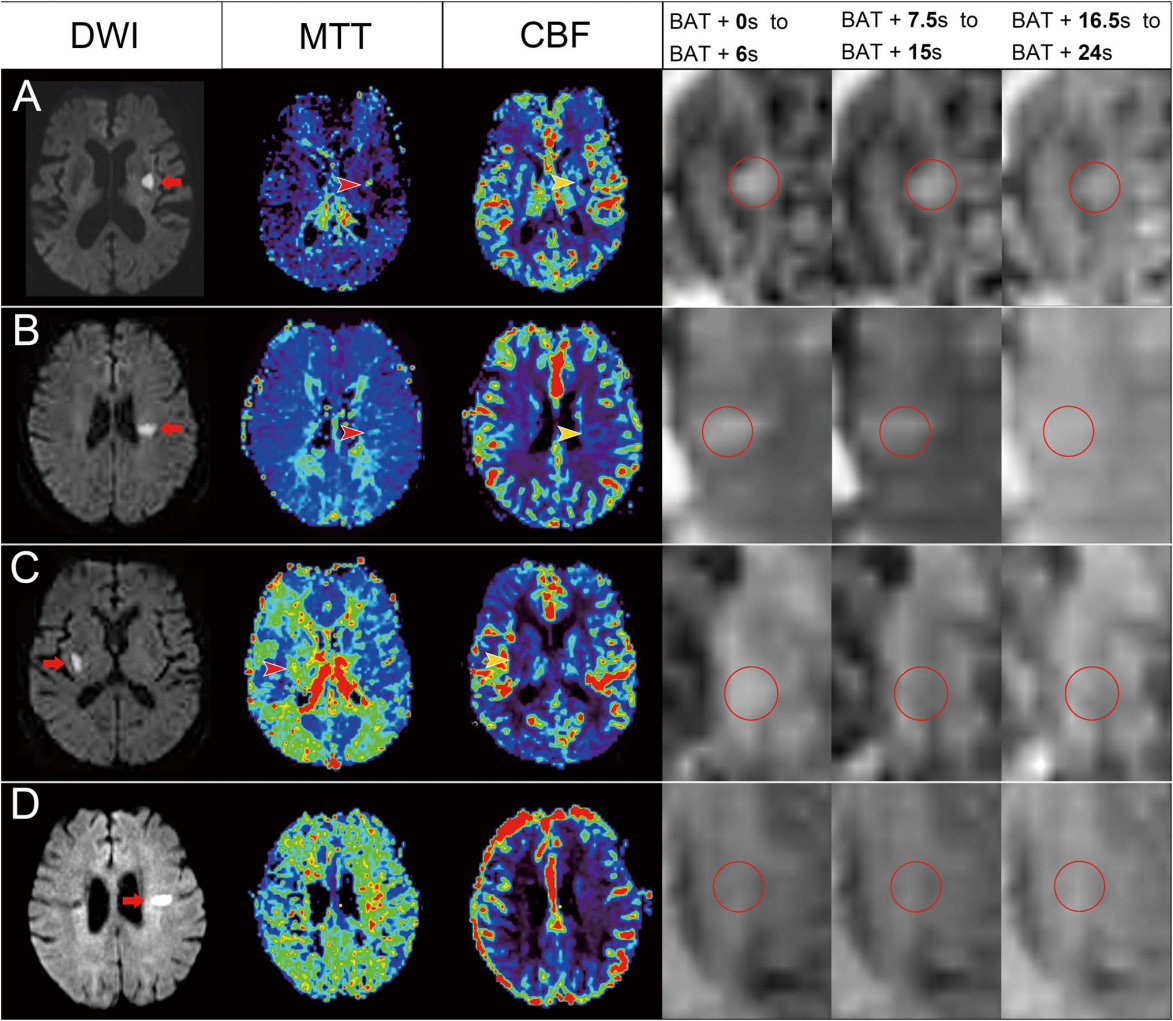
Illustration of different perfusion patterns in the represented patients. (**A**) An 89-year-old woman who had acute infarction in the left basal ganglia (arrow) on diffusion weighted imaging (DWI), with prolonged mean transit time (MTT, red arrowhead) and reduced cerebral blood flow (CBF, yellow arrowhead). The dynamic perfusion imaging showed a perfusion defect without collateral flow, which was classified as a hypoperfusion pattern. (**B**) A 76-year-old woman who had acute infarction in the left centrum semiovale on DWI (arrow) and a perfusion defect on MTT (red arrowhead) and CBF (yellow arrowhead). She was classified as having a hypoperfusion pattern, because of the delayed collateral flow without complete filling. (**C**) An 87-year-old man had acute infarction in the right basal ganglia on DWI (arrow) and a perfusion defect on MTT (red arrowhead) and CBF (yellow arrowhead). He was classified as having compensated perfusion because there was retrograde collateral flow with complete contrast filling. (**D**) An 86-year-old woman had acute infarction in the centrum semiovale on DWI (arrow) without visible perfusion defects. She was classified as having a normal perfusion pattern because of the normal perfusion

The flow patterns were classified as normal perfusion, compensated perfusion, or hypoperfusion (Fig. 1). Patients were classified as normal perfusion when the contrast was completely filled within 6 s plus bolus arrival time (BAT) and patients were classified as compensated perfusion if the contrast was completely filled for > 6 s plus BAT. However, they were classified as hypoperfusion if there was incomplete contrast filling or no contrast filling through the sequential DSC perfusion images.

**Fig. 2:**
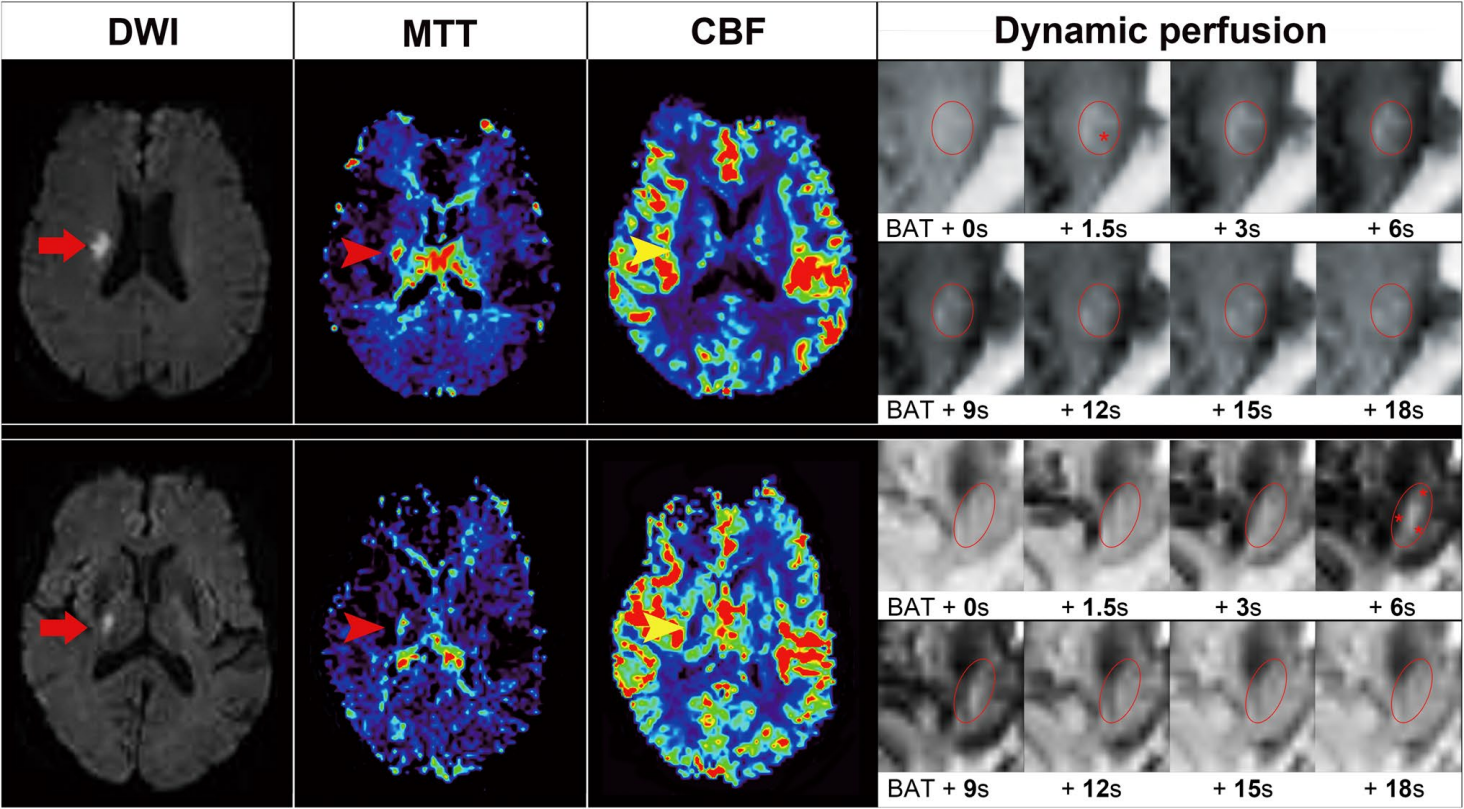
Illustration of patients with anterograde and retrograde collaterals. (**A**) A 76-year-old man had acute infarction in the right centrum semiovale (arrow) on diffusion weighted imaging (DWI), with prolonged mean transit time (MTT, red arrowhead) and reduced cerebral blood flow (CBF, yellow arrowhead). The dynamic perfusion imaging showed a perfusion defect (red circle) with anterograde collateral flows (star), which was present as a decreased signal in the center. (**B**) A 79-year-old man had acute infarction in the right basal ganglia on DWI (arrow) and a perfusion defect on MTT (red arrowhead) and CBF (yellow arrowhead). Dynamic perfusion imaging showed a perfusion defect (red circle) with retrograde collaterals (star), which was present as a decreased signal at the periphery of the circle (star)

**Fig. 3:**
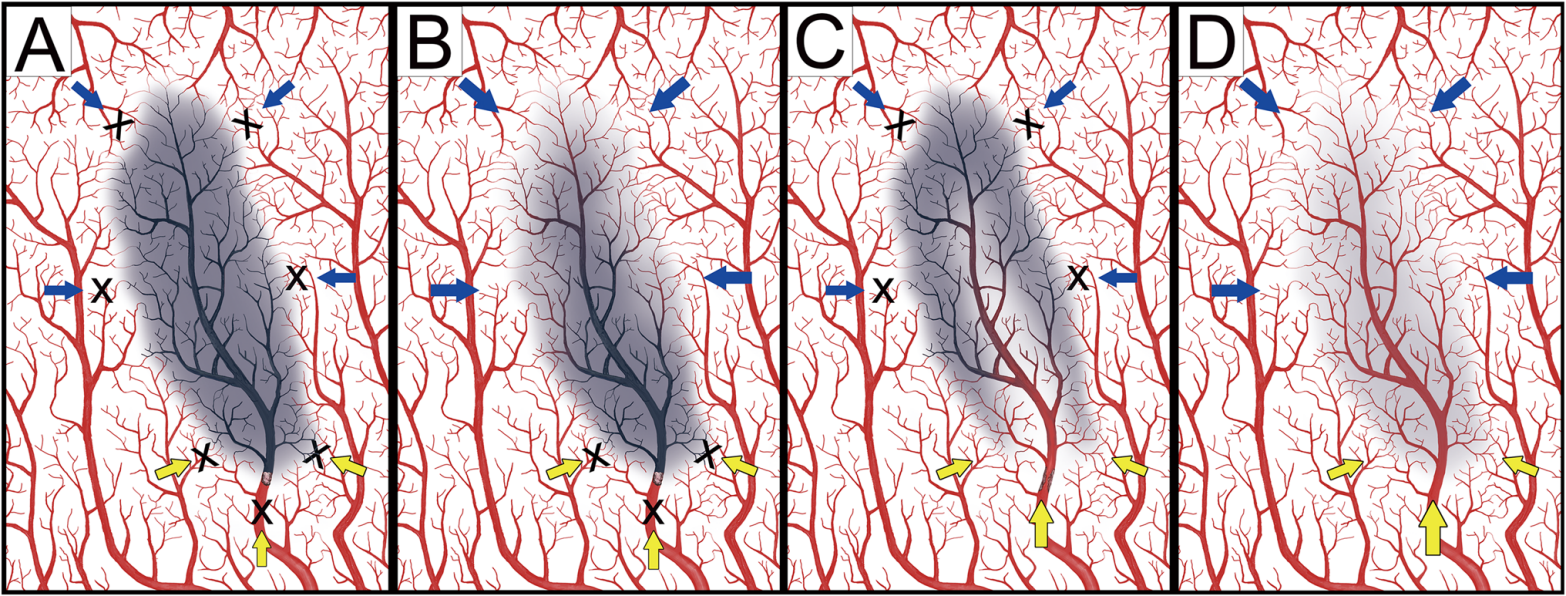
Illustration of different perfusion patterns with or without anterograde collaterals. (**A**) The retrograde (blue arrows) and anterograde (yellow arrows) were markedly decreased and a hypoperfusion pattern was observed. (**B**) The anterograde collaterals were significant reduced and perfusion depended on the retrograde collaterals. This was observed in both hypoperfusion and compensated perfusion patterns. (**C**) The anterograde collateral was prominent and this pattern was observed more often in compensated perfusion and less often in hypoperfusion patterns. (**D**) Both anterograde and retrograde collaterals were present, representing a normal perfusion pattern. This illustration was modified from the figure by Rudilosso et al. [14]

Evaluation of the development of anterograde or retrograde collaterals was performed for all patients first. Anterograde collateral flow was defined as when the filling contrast appeared inside. However, it was classified as retrograde collateral flow if the contrast filled from outside to inside in continuous images (Fig. 2). Patients with normal perfusion patterns were classified as having both anterograde and retrograde collaterals because the cerebral perfusion was almost the same as that on the normal contralateral side. However, patients with normal perfusion patterns did not have visible perfusion defects suggesting spontaneous recanalization so that they were not selected for further analysis of collateral patterns. Evaluation of the development of anterograde or retrograde collaterals was then performed for patients with compensated perfusion or hypoperfusion (Table 2). Figure 3 illustrates the perfusion patterns with correlation to anterograde and retrograde collaterals.

### Neuroimaging Features of SVD and BAD

Neuroimaging features of SVD and their topographic distribution were analyzed, including white matter hyperintensities (WMHs), cerebral microbleeds (CMBs), enlarged perivascular spaces (EPVSs), and lacunes [1]. WMHs were scored using a 4-point scale to assess the degree of white matter changes in the periventricular areas and basal ganglia [18]. The presence of CMBs was classified into three locations according to the Microbleed Anatomical Rating Scale (MARS): lobar (supratentorial cortical and subcortical regions), deep (basal ganglia, thalamus, internal capsule, external capsule, corpus callosum, deep and periventricular white matter), and infratentorial (brainstem and cerebellum) areas [19]. EPVSs were rated on axial T2-weighted imaging using a validated visual rating scale at the level of the centrum semiovale and basal ganglia, respectively [20]. The rating of the EPVS was as follows: 0 = no EPVS, 1 = 1–10 EPVSs, 2 = 11–20 EPVSs, 3 = 21–40 EPVSs, and 4 =  > 40 EPVSs. The presence of lacunes was classified into two locations, including the centrum semiovale and deep (basal ganglia, thalamus and brainstem) areas. BADs were defined when they were visible in four or more of the axial MRI cuts in the lenticulostriate territory, or infarcts that extended from the basal surface of the pons [3, 21].

### Statistical Analyses

Statistical analyses were performed using the Statistical Package for the Social Sciences (SPSS) statistical software (version 18, Chicago, IL, USA). The Kolmogorov–Smirnov test was used to examine the normalization of continuous variables. Continuous variables with normal distribution were presented as mean ± standard deviation (SD and non-normal variables were reported as median (interquartile range [IQR]). Means of 2 and 3 normally distributed continuous variables were compared by independent samples Student’s *t* test and one-way analysis of variance respectively. Mann–Whitney *U* test and Kruskal–Wallis test were used respectively to compare means of 2 and 3 groups of variables not normally distributed. Categorical data was analyzed using Fisher’s exact test or Pearson’s chi-square test, when appropriate. If significant differences were found between the three perfusion patterns by Kruskal–Wallis test or Pearson’s chi-square test, multiple comparisons between three groups were conducted and the significance values had been adjusted by the Bonferroni correction. Inter-rater reliability between two raters for the rating of neuroimaging markers was judged by Cohen’s kappa values.

## Results

A total of 385 patients with suspected stroke who underwent MRI studies within 24 h of symptom onset were evaluated in the current study. A total of 34 patients were unable to finish their MRI scan and 67 did not show any diffusion restriction lesions in their DWIs. Of the remaining 284 patients, 131 had acute SSS with an axial diameter ≤ 20 mm. There were 11 patients with relevant artery stenosis > 50% and 17 patients with perfusion imaging unsuitable for analysis, who were excluded. A total of 103 patients were recruited into the present study, with a median age of 71 (61–77) years and a median NIHSS score of 4 (3–6) on arrival. The median duration from stroke onset to MRI was 16.7(10.5–22.4) hours and 21 (20%) patients had brainstem strokes. There were 100 (97%) patients who presented with classical lacunar syndromes, including 29 (28%) with pure motor, 21 (20%) with sensorimotor, 24 (23%) with ataxic hemiparesis, 21 (20%) with dysarthria-clumsy hand, and 5 (5%) with pure sensory lacunar syndromes. There were 6 (6%) patients with coexistent atrial fibrillation, while 22 patients (21%) had END. The baseline characteristics, imaging findings, and outcomes as well as their association with perfusion defects, are shown in Table 1. Illustrations of different perfusion patterns are shown in Fig. 1. The Cohen’s Kappa coefficients for pattern classification agreement between the two stroke neurologists were 0.70 for perfusion patterns, 0.72 for anterograde collaterals, and 0.73 for retrograde collaterals.

**Table 1:** Correlations between perfusion patterns and baseline characteristics, imaging findings, and outcomes

Characteristic	Normal perfusion *N* = 25	Compensated perfusion *N* = 31	Hypoperfusion *N* = 47	*p* value
Age, median (IQR)	71 (63–79.5)	71 (60–77)	70 (61–78)	0.828
Female gender, n (%)	12 (48%)	12 (39%)	19 (40%)	0.758
**Stroke risk factors**				
Diabetes mellitus, n (%)	13 (52%)	16 (52%)	31 (66%)	0.348
Hypertension, n (%)	21 (84%)	25 (81%)	43 (91%)	0.362
Hypercholesterolemia, n (%)	14 (56%)	14 (45%)	29 (62%)	0.355
Coronary artery disease, n (%)	2 (8%)	0 (0%)	3 (6%)	0.308
Atrial fibrillation, n (%)	2 (8%)	2 (6%)	2 (4%)	0.799
Previous antiplatelet use, n (%)	4 (16%)	2 (6%)	8 (17%)	0.379
**Vital signs and laboratory data**				
SBP (mmHg), mean ± SD	173 ± 35	179 ± 34	186 ± 33	0.283
DBP (mmHg), mean ± SD	98 ± 19	102 ± 16	104 ± 20	0.424
Sugar (mg/dL), median (IQR)	132 (114–161)	145 (120–197)	139 (111–207)	0.345
Creatinine clearance (cc/min), mean ± SD ^**a**^	62 ± 22	73 ± 31	73 ± 28	0.215
Total cholesterol(mg/dL), mean ± SD	182 ± 37	180 ± 41	188 ± 43	0.698
**Stroke information**				
Baseline NIHSS, median (IQR)	3 (2–4.5)	4 (4–6)	5 (3–6)	0.048
NIHSS score on 3^rd^ day, median (IQR)	2 (1–4)	3 (2–6)	4 (3–7)	0.002
END (NIHSS > 2), n (%)	0 (0%)	7 (23%)	15 (32%)	0.007
Initial infarct volume(ml), median (IQR)	0.39 (0.23–0.78)	0.81 (0.6–1.47)	1.01 (0.44–2.35)	< 0.001
Final infarct volume (ml), median (IQR)	0.85 (0.38–1.47)	1.06 (0.70–2.14)	1.80 (0.81–2.64)	0.029
mRS at 3 months, median (IQR)	1 (0–2)	1 (0–2)	1 (0–3)	0.412
mRS ≧ 3 at 3 months, n (%)	6 (24.0%)	6 (19.4%)	17 (36.2%)	0.235
**Neuroimaging information**				
Onset-MRI duration (hour), median (IQR)	16.0 (9.9–21.1)	14.8 (6.8–22.3)	19.4 (13.1–23.0)	0.199
Brainstem, n (%)	9 (36%)	2 (6%)	10 (21%)	0.024
Branch atheromatous disease, n (%)	6 (24%)	15 (48%)	26 (55%)	0.037
rCBF, median (IQR)	1.04 (0.93–1.16)	0.76 (0.52–0.89)	0.63 (0.43–0.81)	< 0.001
rCBV, median (IQR)	1.01 (0.91–1.20)	0.92 (0.76–1.05)	0.77 (0.56–0.97)	0.001
rMTT, median (IQR)	1.01 (0.97–1.12)	1.72 (1.22–2.18)	1.51 (1.12–2.26)	< 0.001
Anterograde collaterals, n (%)	25 (100%)	21 (68%)	19 (40%)	< 0.001
Retrograde collaterals, n (%)	25 (100%)	26 (84%)	31 (66%)	0.002
WMH scores of periventricular area, median (IQR)	1 (1–2)	1 (1–2)	1 (1–2)	0.872
WMH scores of basal ganglia, median (IQR)	1 (0–2)	2 (1–2)	2 (1–2)	0.068
EPVS scores of centrum semiovale, median (IQR)	2 (2–2)	2 (2–2)	2 (2–3)	0.890
EPVS scores of basal ganglia, median (IQR)	2 (1–2)	2 (1–3)	2 (2–2)	0.171
Lacune at centrum semiovale, n (%)	5 (20.0%)	8 (25.8%)	11 (23.4%)	0.877
Lacune at basal ganglia, n (%)	14 (56.0%)	15 (48.4%)	30 (63.8%)	0.398
CMBs at lobar areas, n (%) ^b^	3 (14%)	6 (24%)	7 (20%)	0.667
CMBs at deep areas, n (%) ^b^	3 (13.6%)	7 (28.0%)	8 (22.9%)	0.487
CMBs at infratentorial areas, n (%) ^b^	5 (22.7%)	4 (16.0%)	5 (14.3%)	0.701

^a^ Creatinine clearance was estimated by the Cockcroft-Gault equation

^b^ CMB data was unavailable in 21 patients

*CMBs*, cerebral microbleeds; *END*, early neurological deterioration; *DBP*, diastolic blood pressure; *IQR*, interquartile range; *MRI*, magnetic resonance imaging; *NIHSS*, National Institutes of Health Stroke Scale; *SBP*, systolic blood pressure; *SD*, standard deviation

On the basis of the perfusion patterns, there were 25 (24%) patients with normal perfusion, 31 (30%) with compensated perfusion, and 47 (46%) with hypoperfusion. There was no significant difference between perfusion patterns for stroke risk factors, vital signs, and laboratory data. However, none of the patients with a normal perfusion pattern had END and they had the smallest initial and final infarction volumes (Table 1). As expected, they also had relatively normal rCBF (1.04), rCBV (1.01), and rMTT (1.01). In contrast, patients with hypoperfusion had the highest rate of early neurological deterioration, and the largest initial and final infarction volumes. They also had the lowest rCBF (0.63, *p* < 0.001) and rCBV (0.77, *P* = 0.001), and the lowest rate of anterograde and retrograde collaterals (19%, *p* < 0.001; 66%, *p* = 0.002). Despite low rCBF (0.76) and prolonged rMTT (1.72) in the compensated perfusion patients, the rCBV (0.92) was relatively normal. In 21 patients with brainstem stroke, compensated perfusion patterns were observed notably less (9.5%) than in patients with normal perfusion (42.9%) or hypoperfusion (47.6%) patterns. Among all SVD features, there were no difference between three perfusion patterns. However, patients with hypoperfusion pattern had higher rate of BAD than those with normal perfusion. For baseline characteristics and imaging findings with *p* values < 0.05 in Table 1, the two-by-two comparisons between three perfusion patterns were shown in supplementary table 1.

In the 78 patients without normal perfusion, 30 patients had anterograde collaterals, 57 patients had retrograde collaterals, and 24 patients had both (Table 2). Patients with anterograde collaterals had a lower rate of hypertension. They also had higher rCBV (0.91 vs. 0.77; *p* = 0.024) and more prolonged rMTT (1.93 vs. 1.34; *p* = 0.016). Anterograde collaterals were significantly associated with a shorter onset-MRI duration (12.6 vs. 20.2 h; *p* = 0.020) and a higher rate of deep CMBs (48.1 vs. 21.2%; *p* = 0.028). Infarction at the brainstem was not associated with anterograde collaterals (3 vs. 23%; *p* = 0.024). Patients with retrograde collaterals were associated with higher systolic blood pressure (187 vs. 171 mmHg; *p* = 0 0.031), higher diastolic blood pressure (106 vs. 96 mmHg; *p* = 0.020), smaller initial infarction volume (0.81 vs. 1.34 ml, *p* = 0.031), and a higher rate of lobar CMBs (30 vs. 0%; *p* = 0.013). However, there was no difference for rCBF, rCBV, and rMTT between patients with and without retrograde collaterals.

**Table 2:** Correlations between the collateral patterns and the baseline characteristics, imaging findings, and outcomes

Characteristics	Anterograde *N* = 30	No anterograde *N* = 48	*p*	Retrograde *N* = 57	No retrograde *N* = 21	*p*
Age, median (IQR)	70 (59.5–75.3)	71 (61–78.8)	0.385	70 (60.5–76.5)	71 (61–78.5)	0.701
Female gender, n (%)	10 (33%)	21 (44%)	0.360	21 (37%)	10 (48%)	0.388
**Stroke risk factors**						
Diabetes mellitus, n (%)	15(50%)	32 (67%)	0.143	33 (58%)	14 (67%)	0.483
Hypertension, n (%)	23 (77%)	45 (94%)	0.039	49 (86%)	19 (90%)	0.721
Hypercholesterolemia, n (%)	17 (57%)	26 (54%)	0.829	31 (54%)	12 (57%)	0.828
Atrial fibrillation, n (%)	2 (7%)	2 (4%)	0.626	4 (7%)	0 (0%)	0.569
Previous antiplatelet use, n (%)	4 (13%)	6 (13%)	1.000	7 (12%)	3 (14%)	1.000
**Vital signs and laboratory data**						
SBP (mmHg), mean ± SD	176 ± 33	188 ± 33	0.143	187 ± 35	171 ± 25	0.031
DBP (mmHg), mean ± SD	103 ± 18	104 ± 19	0.828	106 ± 18	96 ± 16	0.020
Sugar (mg/dL), median (IQR)	137 (117–174)	157 (118–214)	0.386	139 (113–213)	154 (127–178)	0.800
Creatinine clearance (cc/min), mean ± SD ^**a**^	76 ± 32	71 ± 27	0.457	74 ± 27	71 ± 34	0.687
Total cholesterol (mg/dL), mean ± SD	188 ± 43	183 ± 41	0.628	184 ± 37	186 ± 54	0.842
**Stroke information**						
Baseline NIHSS, median (IQR)	4 (3–6)	5 (3–6)	0.520	5 (3.5–6)	4 (2.5–7)	0.833
NIHSS score on 3^rd^ day, median (IQR)	3.5 (2–6)	4 (2.25–6)	0.446	4 (2.5–6)	4 (2–6)	0.382
END (NIHSS > 2), n (%)	8 (27%)	14 (29%)	0.811	19 (33%)	3 (14%)	0.155
Initial infarct volume (ml), median (IQR)	1.0 (0.55–1.78)	0.96 (0.48–1.73)	0.869	0.81 (0.43–1.45)	1.34 (0.62–3.2)	0.031
Final infarct volume (ml), median (IQR)	1.52 (0.67–3.00)	1.21 (0.79–2.47)	0.728	1.27 (0.72–2.49)	1.28 (0.82–3.35)	0.452
mRS at 3 months (IQR)	1 (0–3)	1 (0–3)	0.755	1 (0–3)	0 (0–3)	0.141
mRS≧3 at 3 months, n (%)	10 (33.3%)	13 (27.1%)	0.556	17 (29.8%)	6 (28.6%)	0.914
**Neuroimaging information**						
Onset-MRI duration (hour), median (IQR)	12.6 (6.8–21.9)	20.2 (13.2–23.0)	0.020	16.7 (10.4–22.4)	21.1 (10.8–23.0)	0.600
Branch atheromatous disease	17 (57%)	24 (50%)	0.566	28 (49%)	13 (62%)	0.316
Brainstem, n (%)	1 (3%)	11 (23%)	0.024	8 (14%)	4 (19%)	0.724
rCBF, median (IQR)	0.69 (0.49–0.95)	0.66 (0.43–0.81)	0.408	0.65 (0.46–0.86)	0.70 (0.39–0.82)	0.787
rCBV, median (IQR)	0.91 (0.77–1.10)	0.77 (0.58–0.97)	0.024	0.84 (0.66–1.04)	0.86 (0.58–0.99)	0.669
rMTT, median (IQR)	1.93 (1.37–2.75)	1.34 (1.12–1.99)	0.016	1.71 (1.12–2.24)	1.52 (1.06–2.35)	0.702
WMH scores of periventricular area (IQR)	1 (1–2)	1 (1–2)	0.888	1 (1–2)	1 (1–2)	0.208
WMH scores of basal ganglia (IQR)	2 (1–2.25)	2 (1–2)	0.931	2 (1–2)	2 (1–2)	0.925
EPVS scores of centrum semiovale (IQR)	2 (1.75–3)	2 (2–3)	0.894	2 (2–3)	2 (1–2.5)	0.356
EPVS scores of basal ganglia (IQR)	2 (1–3)	2 (1–2)	0.420	2 (1–3)	2 (1–2)	0.379
Lacune at centrum semiovale, n (%)	7 (23.3%)	12 (25.0%)	0.868	15 (26.3%)	4 (19.0%)	0.507
Lacune at basal ganglia, n (%)	16 (53.3%)	29 (60.4%)	0.538	33 (57.9%)	12 (57.1%)	0.952
CMBs at lobar areas, n (%) ^b^	9 (33%)	4 (12%)	0.063	13 (30%)	0 (0%)	0.013
CMBs at deep areas, n (%) ^b^	13 (48.1%)	7 (21.2%)	0.028	16 (36.4%)	4 (25.0%)	0.541
CMBs at infratentorial area, n (%) ^b^	4 (14.8%)	5 (15.2%)	1.000	8 (18.2%)	1 (6.3%)	0.422

^a^ Creatinine clearance was estimated by the Cockcroft-Gault equation

^b^ CMB data was unavailable in 18 patients

*CMBs*, cerebral microbleeds; *END*, early neurological deterioration; *DBP*, diastolic blood pressure; *IQR*, interquartile range; *MRI*, magnetic resonance imaging; *NIHSS*, National Institutes of Health Stroke Scale; *SBP*, systolic blood pressure; *SD*, standard deviation

The analysis of CMBs with correlation to the baseline characteristics and imaging findings was shown in the supplemental Table 2. Patients with lobar CMBs had higher NIHSS scores on the 3rd day (5 vs. 3.5, *p* = 0.020), higher mRS at 3 months (3 vs. 1, *p* = 0.002), higher rate of mRS ≧ 3 at 3 months (56.3 vs. 25.8%, *p* = 0.019), higher rate of BAD (68.8 vs. 39.4%, *p* = 0.034), and higher rate of retrograde collaterals (100 vs. 66%, *p* = 0.013).

## Discussion

The current study demonstrates that there are different hemodynamic compromise and microvascular collaterals in acute SSI. Anterograde and retrograde collaterals may play a critical role in maintaining cerebral perfusion, while perfusion compromise was closely associated with the development of END and final infarct size.

Acute SSI is caused by the occlusion of penetrating arteries, which have been long regarded as independent vessels with less interterritorial anastomoses [6, 22]. However, previous studies observed connections at the precapillary arteriole level among different vascular territories and some anastomosis at proximal perforators [8, 9, 23]. In addition, significant velocity reactivity was measured in both the semioval center and basal ganglia in ultra-high field strength MRIs [24], suggesting an autoregulation of small perforating arteries. An animal model also indicated that arteriolo-arteriolar anastomosis plays a major role in supporting the active dilation of the penetrating arterioles compensating a significant amount of blood to the ischemic region [25]. Our study supports previous findings of compensated collateral flow by Rudilosso et al. [14] and provides indirect evidence for interterritorial connections between penetrating arteries. Given the findings of collaterals and perfusion compromises of different degrees, the penumbral hypothesis may be applied for the microvascular circulation.

In acute large steno-occlusive stroke, both anterograde and retrograde collateral perfusion may account for target territory perfusion and complex intercorrelations may exist between them that determine clinical outcomes and treatment responses. Analysis of the Warfarin–Aspirin Symptomatic Intracranial Disease trial data revealed that good retrograde collaterals were a protective factor against ipsilateral recurrent stroke in patients with severe (70–99%) intracranial atherosclerotic stenosis (ICAS), but a risk factor for recurrent stroke in those with moderate (50–69%) ICAS [26]. These findings may be explained by a later study, which showed that cerebral perfusion was significantly more dependent on anterograde residual flow in moderate MCA stenosis but significantly more dependent on leptomeningeal retrograde collateral flow in severe stenosis, when anterograde residual flow was not enough [27]. Moreover, another study using arterial spin labeling MRI perfusion revealed that early-arriving anterograde flow plays an important role in maintaining perfusion of the target downstream territory in patients with MCA stenosis [28].

Like steno-occlusive disease, our study suggests that anterograde collaterals may play a crucial role in maintaining adequate microvascular perfusion after acute SSI, given that patients with hypoperfusion had lower rates of anterograde collaterals. Anterograde collaterals may come directly from partially occlusive arteries or indirectly from anastomosis of the proximal penetrating arteries. However, there are fewer proximal anastomosis from basilar arteries and MCA [8], so the anterograde collaterals more likely come from partially occlusive arteries. This may also explain why 25 (24%) patients had normal perfusion patterns in the infarct areas; this was probably due to recanalization of the previously partially occluded vessels. Anterograde collaterals were associated with shorter onset-MRI duration and the reason for this observation could be that partially occluded vessels with anterograde collaterals achieved spontaneous recanalization as time passed but completely occluded vessels without anterograde collaterals did not, even after a longer onset-MRI time. However, our study did not repeat MRIs to prove our inference.

Patients with retrograde collaterals were associated with a smaller initial infarct volume, suggesting a protective effect or an existence of penumbra by the retrograde collaterals. As time passed, the followed infarct volume increased because of no recanalization. However, the perfusion parameters were not different between patients with or without retrograde collaterals. One reason is that the anterograde collaterals play the major role in maintaining the tissue perfusion. Another reason, which we think is more likely to be true, is the methodological limitation that the margin of retrograde collaterals is hard to delineate in such a small perfusion defect and the applied ROI did not include the peripheral area with less severely impaired perfusion so that perfusion parameters did not show the difference.

A previous study by Rudilosso et al. reported that systolic blood pressure was higher in patients with sustained hypoperfusion and we also noticed a trend of higher systolic blood pressure in patients with hypoperfusion or in patients without anterograde collaterals. In contrast, retrograde collaterals were associated with higher systolic and diastolic blood pressure. Patients without anterograde collaterals also had a higher percentage of hypertension. Several previous studies revealed that hypertension increases shear stress, endothelial dysfunction, and large artery stiffness that transmits pulsatile flow to the cerebral microcirculation [29]. Hypertension also contributes to structurally smaller lumen of collateral vessels, further reducing the capacity of autoregulation and collateral flow [29, 30]. Therefore, hypertension is considered to have a long-term negative impact on the development of microvascular collaterals in acute SSI. We presume that the absence of anterograde collaterals augments the autoregulation via the elevation of blood pressure, which may increase the retrograde collaterals.

The topographic distribution of SVD imaging markers has been reported to be associated to different pathologies of SVD [2]. Cerebral amyloid angiopathy has been associated with CMBs in the lobar areas [31, 32], WMHs in periventricular areas [33, 34], and EPVSs in the centrum semiovale [35, 36], whereas hypertensive arteriopathy has been linked to CMBs, WMHs, and EPVSs in the basal ganglia [31–36]. Cerebral amyloid angiopathy is more prevalent in the cortical and leptomeningeal vessels and hypertensive arteriopathy affects less specifically but predominantly deep perforating arteries. In this study, anterograde collateral flow was associated with CMBs in deep areas, suggesting a correlation with underlying hypertensive vasculopathy [31, 32], whereas retrograde collateral flow was associated with CMBs in lobar areas, suggesting a correlation with underlying amyloid angiopathy from leptomeningeal vessels [32, 37]. We think it is more likely that hypertensive vasculopathy affects larger perforators and determines the existence of anterograde collaterals. By contrast, cerebral amyloid angiopathy affects smaller leptomeningeal vessels and determines mainly the retrograde collaterals [2]. However, further research is obviously required to investigate the correlations between collaterals and underlying pathologies.

Infarction at the brainstem was less associated with compensated perfusion and anterograde collaterals. These findings may be explained by less autoregulation or fewer proximal anastomosis of the penetrating arteries from the basilar artery [8]. BAD was associated with fewer normal perfusion patterns. This could be because occluded atherosclerotic plaques may achieve spontaneous recanalization less often due to their larger size and dense composition.

There were several limitations to the present study. First, we excluded patients receiving thrombolytic therapy or thrombectomy so that the timing of MRI was beyond the time window of hyperacute stroke. The measurement in our study may not represent the hemodynamic status in hyperacute stage after SSI. In addition, a single measurement of perfusion status may not fully delineate the dynamic changes. Patients with transient symptoms but with SSI were not enrolled and this may have caused the effects of collateral flow to be underestimated. Because of the restricted spatial resolution of perfusion MRI and the susceptibility of bone artifacts, especially in the posterior fossa, tiny perfusion defects and collaterals may have been missed due to these methodological limitations. Moreover, the number of patients included in the study was relatively small. Despite these limitations, our study still provides valuable information that improves current understanding of the hemodynamic status and collateral flow in acute SSI.

In conclusion, this study revealed that there are different microvascular collaterals in acute SSI that determine the variable perfusion compromise and have an impact on clinical outcomes. Both anterograde and retrograde collaterals may play a critical role in maintaining cerebral perfusion. These findings support the penumbral hypothesis for microvascular vascularity in acute SSI and potential pharmacotherapy for recanalization. Further studies are warranted to verify these findings and to investigate effective treatments for the prevention of END and to improve clinical outcomes.

## Supplementary Information

Below is the link to the electronic supplementary material.

Supplementary file1 (DOCX 35 KB)

## Data Availability

The datasets during the current study are available from the corresponding author on reasonable request. The protocols of these studies were approved by the Institutional Review Board of Chang Gung Memorial Hospital (102-4491A3 and 201700668B0). All procedures performed in studies involving human participants were in accordance with the ethical standards of the institutional and/or national research committee and with the 1964 Helsinki Declaration and its later amendments or comparable ethical standards. Informed consent was obtained from all individual participants.
