# Supplementary material for: Perfusion Defects and Collateral Flow Patterns in Acute Small Subcortical Infarction: a 4D Dynamic MRI Study
Source: Transl Stroke Res. 2021 Oct 14;13(3):399–409. doi: 10.1007/s12975-021-00953-x (PMC9046333; doi:10.1007/s12975-021-00953-x)
Supplement: Supplementary file 1 — Supplementary file1 (DOCX 35 KB) [file 12975_2021_953_MOESM1_ESM.docx]

**Supplementary Information for**

**Perfusion defects and** **collateral flow patterns in acute** **small subcortical infarction:** **a 4D dynamic MRI study**

**Authors:**

Yen-Chu Huang^1^, MD; Jiann-Der Lee^1^, MD, PhD; Yi-Ting Pan^1^, MD; Hsu-Huei Weng^2^, MD, MPH, PhD; Jen-Tsung Yang^3^, MD, PhD; Leng-Chieh Lin^4^, MD; Yuan-Hsiung Tsai^2^*, MD, PhD

Department of Neurology^1^, Department of Diagnostic Radiology^2^, Department of Neurosurgery^3^ and Department of Emergency Medicine^4^

Chang Gung Memorial Hospital at Chiayi, Chang-Gung University College of Medicine, Taiwan

**Corresponding author**:

Yuan-Hsiung Tsai

Department of Diagnostic Radiology, Chang Gung Memorial Hospital at Chiayi, Chang-Gung University College of Medicine, Taiwan

No. 6 West Chia-Pu Road, Putz City, Chiayi County, Taiwan

E-mail: russell.tsai@gmail.com

Contents of this supplementary information file include:

- Supplementary Table 1
- Supplementary Table 2

**Supplementary table 1**

Two-by-two comparisons between three perfusion patterns for baseline characteristics and imaging findings.

| **Characteristic** | Normal perfusion vs. compensated perfusion  *p* | Normal perfusion vs. hypoperfusion  *p* | Compensated perfusion vs. hypoperfusion  *p* |
| --- | --- | --- | --- |
| **Stroke information** |  |  |  |
| Baseline NIHSS | 0.098 | 0.014 | 0.482 |
| NIHSS score on 3^rd^ day | 0.075 | 0.001* | 0.102 |
| END (NIHSS>2) | 0.013* | 0.002* | 0.370 |
| Initial infarct volume | 0.002* | <0.001* | 0.454 |
| Final infarct volume | 0.143 | 0.008* | 0.197 |
| **Neuroimaging information** |  |  |  |
| Brainstem | 0.006* | 0.177 | 0.076 |
| Branch atheromatous disease | 0.061 | 0.011* | 0.549 |
| Relative CBF | <0.001* | <0.001* | 0.171 |
| Relative CBV | 0.117 | 0.001* | 0.046 |
| Relative MTT | <0.001* | <0.001* | 0.665 |
| Anterograde collateral | 0.002* | <0.001* | <0.001* |
| Retrograde collateral | 0.035 | 0.001* | 0.081 |

Two-by-two comparisons were conducted using the chi-square test or Kruskal-Wallis test, and the significance values have been adjusted by the Bonferroni correction for multiple tests.

*: A *p* value <0.017 (α/n=0.05/3), indicating a statistically significant difference.

Abbreviations: CBF=cerebral blood flow; CBV= cerebral blood volume; END=early neurological deterioration; MTT= mean transit time; NIHSS=National Institutes of Health Stroke Scale.

**Supplementary table 2**

Correlations between the cerebral microbleeds and the baseline characteristics and imaging findings.

| **Characteristics** | **Any CMBs (+)**  **N= 29** | **Any CMBs (-)**  **N= 53** | ***p*** |  | **Deep CMBs (+)**  **N= 25** | **Deep CMBs (-)**  **N= 57** | ***p*** |  | **Lobar CMBs (+)**  **N= 16** | **Lobar CMBs (-)**  **N= 66** | ***p*** |
| --- | --- | --- | --- | --- | --- | --- | --- | --- | --- | --- | --- |
| Age, median (IQR) | 69 (62–69) | 71 (60–75.5) | 0.482 |  | 67 (61.5–79) | 71 (60.5–77) | 0.984 |  | 75.5 (64–85.5) | 70 (61–74.25) | 0.072 |
| Female gender, n (%) | 9 (31.0%) | 26 (49.1%) | 0.115 |  | 7 (28.0%) | 28 (49.1%) | 0.075 |  | 5(31.3%) | 30 (45.5%) | 0.303 |
| **Stroke risk factors** |  |  |  |  |  |  |  |  |  |  |  |
| Diabetes mellitus, n (%) | 17 (58.6%) | 30 (56.6%) | 0.860 |  | 15 (60.0%) | 32 (56.1%) | 0.745 |  | 8 (50.0%) | 39 (59.1%) | 0.510 |
| Hypertension, n (%) | 25 (86.2%) | 45 (84.9%) | 1.000 |  | 22 (88.0%) | 48 (84.2%) | 0.748 |  | 14 (87.5%) | 56 (84.8%) | 1.000 |
| Hypercholesterolemia, n (%) | 17 (58.6%) | 26 (49.1%) | 0.407 |  | 16 (64.0%) | 27 (47.4%) | 0.165 |  | 6 (37.5%) | 37 (56.1%) | 0.182 |
| Atrial fibrillation, n (%) | 1 (3.4%) | 3 (5.7%) | 1.000 |  | 1 (4.0%) | 3 (5.3%) | 1.000 |  | 0 (0%) | 4 (6.1%) | 0.581 |
| Previous antiplatelet use, n (%) | 5 (17.2%) | 7 (13.2%) | 0.621 |  | 5 (20.0%) | 7 (12.3%) | 0.363 |  | 2 (12.5%) | 10 (15.2%) | 1.000 |
| **Vital signs and laboratory data** |  |  |  |  |  |  |  |  |  |  |  |
| SBP (mmHg), mean ± SD | 182 ± 32 | 178 ± 33 | 0.616 |  | 184 ± 33 | 178 ± 33 | 0.413 |  | 179 ± 32 | 180 ± 33 | 0.908 |
| DBP (mmHg), mean ± SD | 107 ± 17 | 98 ± 18 | 0.034 |  | 107 ± 17 | 98 ± 18 | 0.056 |  | 106 ± 16 | 100 ±18 | 0.204 |
| Sugar (mg/dL), median (IQR) | 136 (117.5–175.5) | 145 (110–180) | 0.920 |  | 136 (119–172) | 145 (110–189) | 0.791 |  | 136 (116.3–169) | 141 (111–187) | 0.626 |
| Creatinine clearance (cc/min), mean ± SD | 68 ± 28 | 70 ± 26 | 0.840 |  | 71 ± 29 | 68 ± 26 | 0.723 |  | 61 ± 29 | 71 ± 26 | 0.169 |
| Total cholesterol (mg/dL), mean ± SD | 189 ± 33 | 183 ± 47 | 0.531 |  | 185 ± 32 | 185 ± 46 | 0.969 |  | 183 ± 37 | 185 ± 44 | 0.862 |
| **Stroke information** |  |  |  |  |  |  |  |  |  |  |  |
| Baseline NIHSS, median (IQR) | 5 (3.5–6) | 4 (2.5–6) | 0.466 |  | 5 (3–6) | 4 (3–6) | 0.714 |  | 5 (4–6.75) | 4 (2.75–6) | 0.200 |
| NIHSS score on 3^rd^ day, median (IQR) | 5 (2.5–7) | 4 (2–6) | 0.178 |  | 5 (2–6) | 4 (2–6) | 0.468 |  | 5 (3.25–9.75) | 3.5 (2–6) | 0.020 |
| END (NIHSS>2), n (%) | 9 (31.0%) | 9 (17.0%) | 0.142 | (%) | 7 (28.0%) | 11 (19.3%) | 0.381 |  | 6 (37.5%) | 12 (18.2%) | 0.094 |
| Initial infarct volume(ml), median (IQR) | 0.87 (0.37–1.86) | 0.73 (0.40–1.28) | 0.401 |  | 1.00 (0.39–1.86) | 0.73 (0.38–1.28) | 0.309 |  | 0.93 (0.45–1.47) | 0.70 (0.39–1.35) | 0.454 |
| Final infarct volume (ml), median (IQR) | 1.89 (0.56–3.19) | 1.06 (0.69–1.84) | 0.120 |  | 1.90(0.56–3.19) | 1.06 (0.69–1.98) | 0.179 |  | 2.10 (0.71–3.21) | 1.06 (0.67–2.02) | 0.111 |
| mRS at 3-months, median (IQR) | 1 (0–3.5) | 1 (0–3) | 0.320 |  | 1 (0–3.5) | 1 (0–3) | 0.758 |  | 3 (1–4) | 1 (0–3) | 0.002 |
| mRS≧3 at 3-months, n (%) | 12 (41.4%) | 14 (26.4%) | 0.164 |  | 10 (40.0%) | 16 (28.1%) | 0.285 |  | 9 (56.3%) | 17 (25.8%) | 0.019 |
| **Neuroimaging information** |  |  |  |  |  |  |  |  |  |  |  |
| Onset-MRI duration (hour), median (IQR) | 16.2 (11.2–21.9) | 19.4 (10.6–22.6) | 0.624 |  | 16.3 (12.4–22.1) | 18.6 (10.4–22.4) | 0.829 |  | 15.8 (12.0–22.8) | 18.5 (10.4–22.3) | 0.810 |
| Branch atheromatous disease | 17 (58.6%) | 20 (37.7%) | 0.069 |  | 15 (60.0%) | 22 (38.6%) | 0.073 |  | 11 (68.8%) | 26 (39.4%) | 0.034 |
| Brainstem, n (%) | 7 (24.1%) | 11 (20.8%) | 0.723 |  | 6 (24.0%) | 12 (21.1%) | 0.767 |  | 3 (18.8%) | 15 (22.7%) | 1.000 |
| rCBF, median (IQR) | 0.67 (0.40–1.01) | 0.80 (0.56–1.04) | 0.200 |  | 0.67 (0.40–1.03) | 0.80 (0.55–1.04) | 0.316 |  | 0.67 (0.54–1.04) | 0.78 (0.49–1.03) | 0.902 |
| rCBV, median (IQR) | 0.85 (0.67–1.01) | 0.97 (0.69–1.05) | 0.731 |  | 0.91 (0.69–1.08) | 0.94 (0.68–0.94) | 0.709 |  | 0.80 (0.67–1.04) | 0.97 (0.70–1.05) | 0.570 |
| rMTT, median (IQR) | 1.77 (1.08–2.56) | 1.38 (1.04–1.84) | 0.204 |  | 1.77 (1.04–2.56) | 1.38 (1.06–1.90) | 0.341 |  | 1.83 (1.08–2.28) | 1.39 (1.03–1.97) | 0.383 |
| Anterograde collaterals, n (%) | 20 (69.0%) | 29 (54.7%) | 0.208 |  | 18 (72.0%) | 31 (54.4%) | 0.134 |  | 12 (75.0%) | 37 (56.1%) | 0.256 |
| Retrograde collaterals, n (%) | 25 (86.2%) | 41 (77.4%) | 0.395 |  | 21 (84.0%) | 45 (78.9%) | 0.765 |  | 16 (100%) | 50 (75.8%) | 0.033 |

The presence of CMBs was classified into three locations according to the Microbleed Anatomical Rating Scale (MARS): lobar, deep and infratentorial areas. Any CMBs (+) indicates the presence of cerebral microbleed in at least one of the lobar, deep or infratentorial areas.

Abbreviations: CMBs=cerebral microbleeds; END=early neurological deterioration; DBP=diastolic blood pressure; IQR=interquartile range; MRI=magnetic resonance imaging; NIHSS=National Institutes of Health Stroke Scale; SBP=systolic blood pressure; SD=standard deviation.
